# Imaging of vertical surface-breaking cracks in concrete members using ultrasonic shear wave tomography

**DOI:** 10.1038/s41598-023-48699-w

**Published:** 2023-12-08

**Authors:** Sai Teja Kuchipudi, Sergey Pudovikov, Herbert Wiggenhauser, Debdutta Ghosh, Ute Rabe

**Affiliations:** 1 CSIR-Central Building Research Institute, Roorkee, 247667 India; 2 Academy of Scientific and Innovative Research, Ghaziabad, 201002 India; 3 Fraunhofer Institute for Nondestructive Testing IZFP, Campus E3.1, Saarbrücken, 66123 Germany; 4 Berlin, Germany

**Keywords:** Engineering, Materials science, Physics

## Abstract

Surface-breaking cracks in concrete pose a significant threat to the durability of built structures. They create pathways for an accelerated moisture intrusion, and the transport of environmental and chemical agents such as chloride ions, which in turn, exacerbate reinforced concrete deterioration. Estimating the extent of crack propagation beneath the surface helps in determining the safety, durability and reliability of a structure. This information can be used to devise appropriate repair methodologies. Developments in Dry Point Contact (DPC)-based ultrasonic arrays have enabled the detection and localization of defects in concrete through high-resolution imaging. This work proposes the use of Half-Skip travel modes in the time-domain pulse-echo data to be deployed for imaging vertical cracks in concrete. In contrast to the standard Synthetic Aperture Focusing Technique (SAFT), which is widely used for ultrasonic imaging of concrete, the Half-Skip Total Focusing Method (HSTFM) configuration uses the target-scattered signals after a reflection from the back wall. The technique has been evaluated on both simulated and experimentally measured ultrasonic data on specimens fabricated with notches and cracks of different depths. Crack sizes were estimated by measuring the length of thresholded reflection signatures obtained in the images. The presented solution using half-skip modes serves as a supplementary technique to standard SAFT, in order to estimate the depth of surface-breaking cracks in concrete.

## Introduction

### Motivation

Concrete is naturally prone to cracking due to fatigue, mechanical and thermal stresses. However, micro-cracks in shallow regions with surface openings less than 0.1 mm are accepted by standard codes^1^. Surface-Breaking Cracks (SBCs) that extend much deeper into concrete further accelerate the deterioration process by creating pathways for the inflow of gas and liquids that lead to corrosion of the steel reinforcement. They also accelerate deterioration in concrete through carbonation and other chemical processes. A reliable assessment of concrete with respect to the presence and size of cracks gives early information for maintenance to achieve its intended service life. Estimating the crack depth (partial/full-depth) without destruction is especially significant for concrete members with single surface exposure such as pavements, dams, bridge girders, and raft foundations.

Assessment of components through Nondestructive Evaluation (NDE) is a sustainable solution of high interest. Among the spectra of available nondestructive methods, elastic wave-based techniques are sensitive to the changes in the mechanical properties of the medium caused by the onset of defects in concrete. The changes in propagation characteristics of ultrasonic waves with varying elastic properties in concrete make them highly suitable for inspections.

### Existing crack characterization techniques

The simplest way to characterize SBCs for their width is through visual examination of the exposed surfaces, as for examples in^2,3^. There are also standards^4,5^ which refer to testing procedures to measure crack width^4^ and analytical approaches^5^. However, quantifying for other interior details, such as inclination and depth of crack penetration, is not possible with visual surface inspections. Drilling cores at regions with visible surface cracks and visually estimating the extent of vertical crack propagation in that core is one of the solutions recommended by testing standards^5,6^. Nondestructive testing methods like the ultrasonic through-transmission testing are one standard way^5,7^ to evaluate for depth of cracks in concrete through measurements in the time of flight of the pulse^8^. There were attempts^9^ using the Impact Echo method^10^ to measure the depth of surface-breaking cracks by identifying the weak frequency components of waves scattered by the tip of cracks.

Later developments^11,12^ in the time-domain methods based on stress waves estimated the crack depth by identifying the first arrival of compressional waves (P-waves) after a reflection from the crack tip. However, these methods remain unreliable, as in addition to the P-waves, other acoustic wave modes are also excited by an impact^13^. To overcome the limitations of P-waves, researchers worked on the application of Rayleigh waves^14–16^ to evaluate the depth of SBCs in concrete. Following a mechanical point impact, surface waves possess much higher energy than P-waves. Their wavelength-dependent transmission characteristics in the near-surface region are sought to be leveraged for estimating the depth of SBCs. However, Rayleigh wave-based inspections are largely hindered by interference due to reflections from boundaries^16^, especially in concrete members with limited dimensions. The penetration depth of Rayleigh waves is approximately equal to one wavelength; thus weaker energy transmission into deeper subsurface regions is another challenge for evaluating full-depth cracks using Rayleigh waves.

The material heterogeneity of concrete causes attenuation, scattering, and mode changes of ultrasonic waves propagating through the medium. The availability of Dry Point Contact (DPC) Shear-Horizontal (SH) transducers^17^ in arrays^18^ has enabled the rapid acquisition of shear wave echoes for tomographic imaging of concrete. It is well known that attenuation by ultrasonic scattering depends on the ratio of the wavelength to the diameter/dimension of the scatterer. Numerical studies^19^ prove that the scattering attenuation of SH waves is less significant at frequencies lower than 50 kHz for commonly used aggregate of size 16 mm in concrete. SH waves are observed to be delivering a high Signal-to-Noise Ratio (SNR) and no mode conversions upon diffraction^20,21^. Shear-Horizontal waves were also applied in metals^22^ for estimating the depth of a Surface-Breaking Crack. In concrete, the scattered pulses are analyzed^23^ for travel-time-based positioning of the crack tip. However, the latest studies attempt to image the crack tip^24–26^ through which the depth can be estimated. One such study^24,26^ proposes the corner echo formed by the crack tip and flat back wall as an indicator to analyze the crack depth. Some other applications with DPC SH-wave arrays include imaging underlying delaminations^27–29^, debonded rebars^27^, and other inclined planar defects^30^ in reinforced concrete.

With multiple transducers simultaneously in action, ultrasonic arrays are more flexible and comprehensive than single-element transducers in terms of the data they produce. The ultrasonic DPC transducers for the inspection of concrete are commercially available as linear and matrix arrays. Usually, they follow the Half Matrix Capture (HMC)^31^ principle for data acquisition^25^. HMC makes use of acoustic reciprocity^32^ to reduce the number of redundant transmissions involved in imaging. Contrary to the Full Matrix Capture (FMC) principle, where all transducer elements consecutively act as transmitters and receivers, in HMC the sequential transmission happens with a single transducer exciting the medium and elements that did not serve as transmitters in previous HMC cycles act as receivers. Furthermore, the transducer, which acted as a transmitter, never acts as a receiver in the same cycle. This reduces the amount of data captured to exactly half of which can be captured with FMC, thus also increasing the speed of measurement and reconstruction.

The post-processing of the ultrasonic array data with the Synthetic Aperture Focusing Technique (SAFT)^33^ is a widely accepted time-domain reconstruction technique in ultrasonic wave-based inspections for the detection of flaws^34,35^, delaminations^27^ and condition assessment of reinforcements^36,37^ in concrete. The application of the SAFT algorithm to FMC or HMC with data from multiple transducer combinations is called the Total Focusing Method (TFM)^31,38,39^.

The SAFT/TFM based ultrasonic devices perform well for the ultrasonic inspection and sizing of defects like delaminations or cracks, which are oriented parallel to the inspection surface^27,30^. Despite this, SAFT/TFM has been reported to deliver poor results in imaging planar defects aligned perpendicular to the surface on which the linear array is placed^30,39^. This accounts for the limitations of an image reconstruction principle, which only considers the directly scattered waves from any object in the imaged volume. In case of inspection of vertical notches in metals^39^, TFM reconstructs only the tip of the notch. Similar observations are found with artificial planar defects embedded in concrete^23,30^.

The introduction of “Half-Skip'' travel modes integrates a reflection at a back wall into the measurement and evaluation. It has recently been shown that one such method, the corner echo formed by the vertical crack plane and a plane back wall, can be used to decide whether a crack in concrete is a full-depth crack^24,26^. In another study, three techniques, SAFT/TFM, Plane-Wave Imaging, and Half-Skip TFM (HSTFM)^30^ were evaluated to find the optimum methodology with respect to defect inclination. Ultrasonic array data measured on a concrete block with artificial planar defects in different inclinations were evaluated. While TFM and Plane-Wave Imaging performed best for defects parallel to the surface or inclined defects, respectively, HSTFM was^30,39^ found to be appropriate for planar vertical defects. However, the technique has not been demonstrated with natural cracks in reinforced concrete. Overall, there have been no existing solutions to image vertical SBCs in reinforced concrete structures. Any new developments in this direction would improve confidence in estimating the extent of the crack’s propagation beneath the surface.

This study addresses the stated problem of imaging vertical SBCs in concrete by implementing Half-Skip travel modes into the reconstruction algorithm of ultrasonic time-domain data. The application of Half-Skip transmission modes for imaging cracks was first developed for metals^39–42^ and later adapted to artificial planar defects in concrete^30^.

The study presented here brings out insights from a detailed assessment of the technique on various defect types (notches and cracks) in both simulated and experimental environments. To study the ultrasonic wave responses on natural cracks, intact reinforced concrete specimens were cracked from the surface under controlled conditions^43^. A commercial SH-wave linear array for concrete with DPC transducers was used for the ultrasonic measurements. Ultrasonic images were reconstructed based on the Half-Skip hypothesis. In addition, this study also addresses the problem of quantifying crack sizes by image-based defect-amplitude thresholding.

## Reconstruction using SAFT/TFM and half-skip TFM

**Figure 1:**
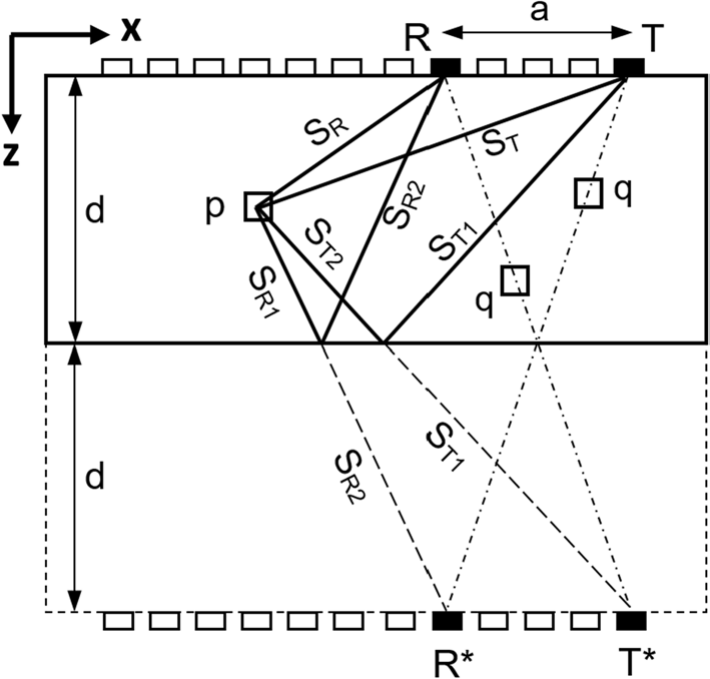
Schematic representation of the sound propagation assumed by TFM and by two cases of half-skip TFM (T*–P–R and T–P–R*).

The Total Focusing Method (TFM) is a standardized^31^ and widely accepted imaging technique, applicable to the pulse-echo data from ultrasonic arrays. The TFM technique operates on the amplitudes of an acoustic pulse excited by transmitter *T*, which gets scattered by the scatterers *p* in the medium and reaches the receiver *R*. The excited pulse is assumed to follow the path *S*_*T*_*–S*_*R*_, as shown in Fig. 1. The SAFT and TFM techniques are based on the assumption that every single point in the discretized volume i.e., the Region of Interest (ROI), is a potential scatterer, which is a source of secondary waves and delivers an acoustic response. As already mentioned above, in the ultrasonic community, the expression “SAFT” is used for the processing of monostatic pulse-echo data, while “TFM” is used for multistatic transmitter–receiver data. Based on the time-of-flight estimations, Eq. (1) gives a mathematical expression for finding the pixel intensity *Ip* corresponding to all such scattering points *p* for any transmitter and receiver (*T – R*) combinations.

1 $${I}_{p}={\sum }_{T=1}^{n} {\sum }_{R=1}^{n} {A}^{TR}\left({t}_{TFM}=\frac{{S}_{T}+{S}_{R}}{V}\right)$$

Here, *T* and *R* are the indices corresponding to the transmitting and receiving sensors,* n* is the total number of the transducers in the array, *A*^*TR*^ is the amplitude of the waveform corresponding to the ultrasonic pulse transmitted between the sensor pairs *T* and *R*, which is a function of propagation time *t*, with a wave velocity *V*. The summed-up amplitudes for all individual scatterers in the ROI over all combinations of *T* and *R* from the Full Matrix Capture (FMC) data form a TFM image. However, as already mentioned above, most of the commercially available ultrasonic array systems for concrete testing do not acquire the whole FMC matrix. Instead, they realize a part of the *T/R* combinations with *T* < *R,* which results in the acquisition of *0.5* × *n* × (*n *− 1) ultrasonic signals from *n* × (*n *− 1) possible for faster and economical operations. This eliminates redundancies in data and is termed as the Half Matrix Capture (HMC)^31^. The pixel intensities with a HMC capture are calculated according to Eq. (2).

2 $${I}_{p}={\sum }_{T=1}^{n-1} {\sum }_{R=T+1}^{n} {A}^{TR}\left({t}_{TFM}=\frac{{S}_{T}+{S}_{R}}{V}\right)$$

The Half-Skip Total Focusing Method (HSTFM) is a modification to the SAFT/TFM. Skipped ultrasonic waves are those that take an indirect insonification path to the scattering point* p* after a single reflection at the back wall. Such skipped pulses, when scattered by a target, create a ‘Half-Skip echo’. The HSTFM uses the information, which is contained in the acoustic waves reflected once on the back wall surface (following Snell's law). It can be assumed that the back wall reflection can happen either on the way between the transmitter *T* and the scatter point *p* or on the way from the point *p* to the receiver *R*, i.e., the pulse follows the path S_T1_–S_T2_–S_R_ or S_T_–S_R1_–S_R2_, respectively, towards the receiver *R*, as illustrated in Fig. 1. The sound propagation path in Half-Skip mode from transmitter *T* to the receiver *R* via the point *p* and the back wall surface is equivalent to the propagation path between the physical sensor *T* or *R* and the imaginary sensor R* or T*, respectively. The sensors marked with a star, R* / T*, are mirrored points of *R / T* with respect to the back wall surface, as shown in Fig. 1.

For a given position of *T* and *R*, the two half-skip paths (T*–p–R and T–p–R*) have different lengths and can be used for image reconstruction. Numerically, the intensities are summed according to the following expressions (Eqs. 3 and 4) for both paths.

3 $${\mathrm{for } \,\, T^{\ast}{\rm -}p{\rm-}R \quad I}_{p}={\sum\limits }_{T=1}^{n-1} {\sum\limits }_{R=T+1}^{n} {A}^{TR}\left({t}_{HSTFM}\frac{{S}_{T1}+{S}_{T2}+{S}_{R}}{V}\right)$$

4 $${\mathrm{for }\,\, T{\rm -}p{\rm -}R^{\ast} \quad I}_{p}={\sum\limits }_{T=1}^{n-1} {\sum\limits }_{R=T+1}^{n} {A}^{TR}\left({t}_{HSTFM}\frac{{S}_{T}+{S}_{R1}+{S}_{R2}}{V}\right)$$

**Figure 2:**
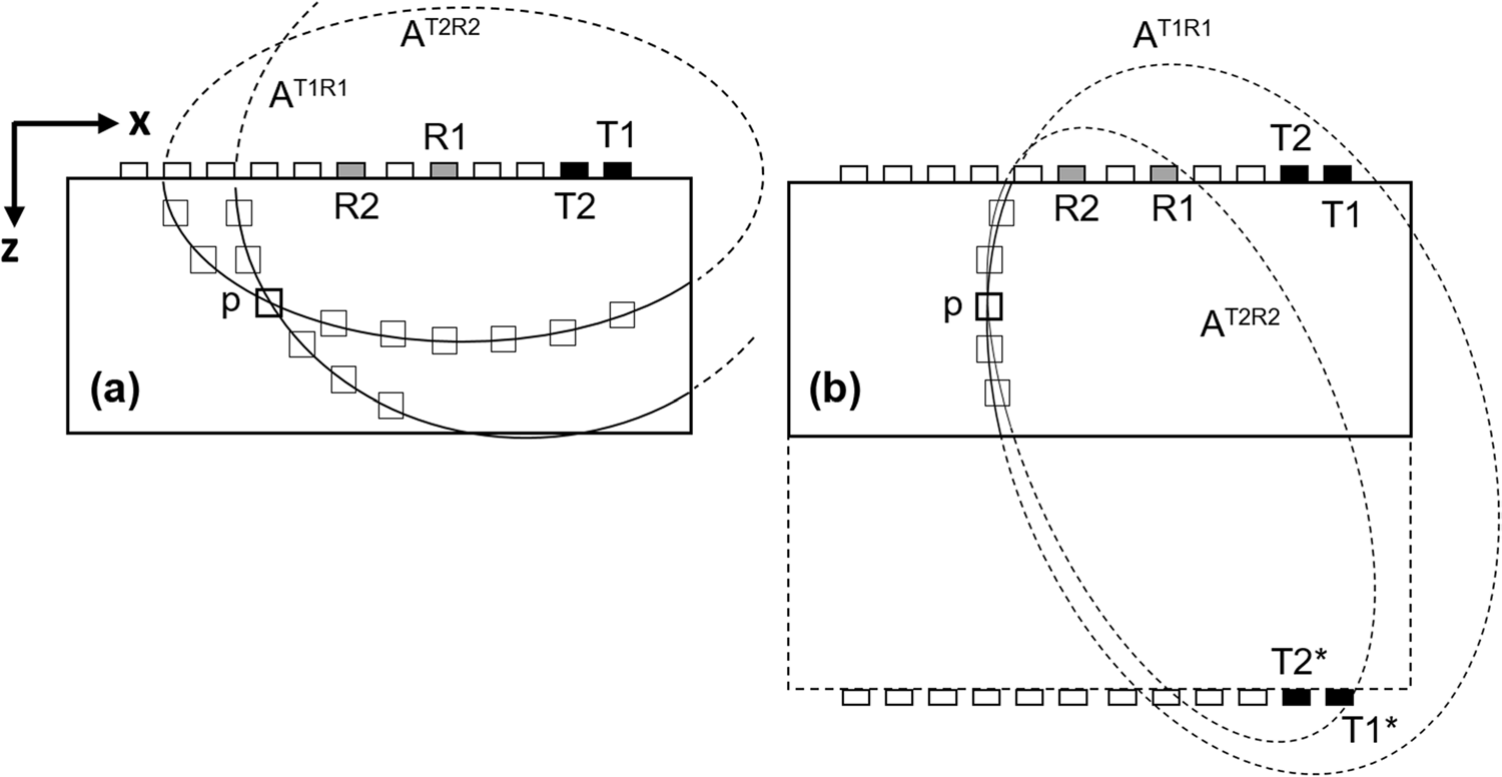
Locus of all points with same propagation time as that of *p* using the (**a**) SAFT/TFM and (**b**) HSTFM. Two transmitter/receiver pairs (*T1, R1*) and (*T2, R2*) were considered. Both the ellipses *A*^*T1R1*^ and *A*^*T2R2*^ intersect in point *p*.

For both techniques, TFM and HSTFM, the locus of points with identical propagation time of one arbitrary transducer–receiver pair (*T–R*) is an ellipse. For SAFT/TFM the focal points (*Ti, Ri*) of the ellipse correspond to the real transmitter and real receiver positions on the inspection surface. In the case of HSTFM, one focal point is in the position of the real array element, and the second focal point is an imaginary source: (Ti*, Ri) or (*Ti, Ri*). Figure 2a shows an example of the projection locus of two transducer-receiver pairs (*T1, R1*) and (*T2, R2*) for TFM and for the (T1*, R1) and (T2*, R2) variant of HSTFM (Fig. 2b).

The travel time corresponding to the respective path to point *p* was chosen. It is a consequence of the SAFT superposition principle that the ellipses intersect in this focal point *p*. In the case of SAFT/TFM (Fig. 2a) the two ellipses intersect with a well-visible angle in point *p* and are clearly separated in all other points. Though the same transducer-receiver pair and the same point *p* were chosen for HSTFM (Fig. 2b), the ellipses are very close to each other in the neighborhood of point *p*. This is caused by the fact that one of the focal points is an imaginary source at the depth *2d*, which leads to a rotation of the ellipses.

The SAFT/TFM/HSTFM reconstructions are based on the superposition of the amplitude data from multiple transmitter–receiver (*T–R*) combinations. The amplitude signals upon summation get either intensified (presence of scatterer) or weaken (absence of scatterer) in the image plane. Such interference of multiple signals from the array improves the SNR. Besides its dependence on the amount of the signals involved, SNR largely depends on the difference in travel time or phase information of the constituent signals undergoing summation. These factors are closely associated with the spatial arrangement of the sensors in the array. For a chosen pair of array transducers, the closeness of the ellipses in Fig. 2b compared to Fig. 2a leads to a smaller phase difference and in consequence to a lower focusing capability.

To theoretically estimate the focusing capability of the HSTFM-based reconstruction, and to compare it with that of the SAFT/TFM, we simulated a case study close to the practical conditions.

As a criterion for the focusing capability of the array in focal point *f* we examine the phase shift for different points *p*, which are close to the considered focal point. The phase shift between the signals, which are received by the points *p* and* f*, is equal to the difference in the corresponding sound paths (see Fig. 1), divided by the wavelength. Since in FMC or HMC data acquisition many combinations of transmitters and receivers are involved, we analyze the maximal difference in the propagation paths for any combination “transmitter–receiver”. By this calculation, we estimate the minimal size of an indication from a scatterer in point *f* for SAFT/TFM and HSTFM procedures. The indication in the reconstructed image is formed by projection and summation of all signals from all transmitters and receivers.

**Figure 3:**
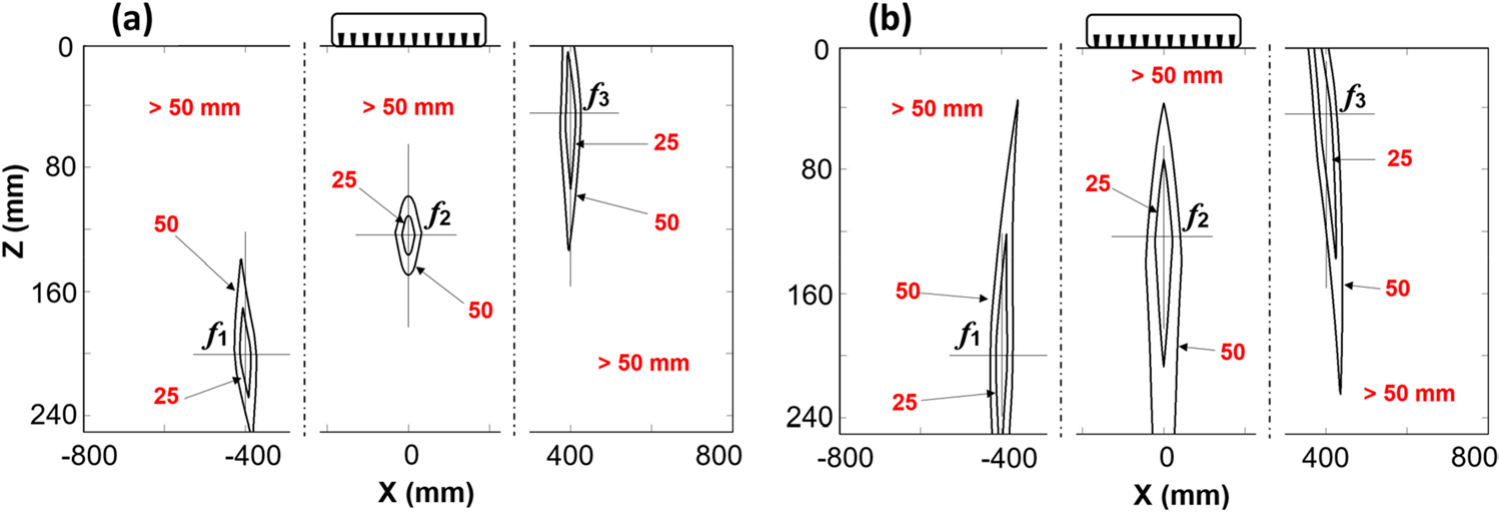
Isolines derived using the techniques (**a**) SAFT/TFM, (**b**) HSTFM showing a constant amount of phase shift Δ*Sp* relative to the three focal points *f*_*1*_, *f*_*2*_ and* f*_*3*_ indicated by three crosses.

A linear ultrasonic array with 12 transducers is assumed to be placed on a 250 mm thick concrete block. Similar to the commercially available ultrasonic arrays for concrete testing, the transducers are separated by a pitch of 30 mm and they follow the HMC for data acquisition. The analysis has been done on a Region of Interest spanning ± 800 mm in both directions of the middle of the sensor array. Three focal points (*f*_*1*_*, f*_*2*_ and* f*_*3*_) in the image plane with the coordinates $({\text{x}},{\text{z}})$ = (− 400, 200), (0, 120) and (400, 50), respectively, were selected for the evaluation. A step of 2 mm along both axes (X and Z) was implemented. The first result is the distribution of the sound path difference with respect to the chosen focal points *f*_*1*_–*f*_*3*_. To associate the calculated path difference values with the focusing capability, we assume the sound velocity in the concrete block as *V* = 2500 m/s. With the operating frequency of 50 kHz we obtain a wavelength of 50 mm. The areas with the sound path differences 25 mm (half a wavelength, or phase shift π) and 50 mm (one wavelength, or phase shift 2π) are marked with isolines in Fig. 3. The chosen value for the velocity fits well with our measured values for the concrete specimens (see “Data collection on reference blocks using the ultrasonic array”).

The area exclusively containing the points which have a path difference not exceeding half of the wavelength λ/2 characterizes an area, in which complete destructive interference cannot happen. The focusing capability reduces with increasing area surrounding a focal point corresponding to a lower degree of maximal phase shift (≤ λ/2), while a smaller area corresponds to a stronger phase shift and finer resolution capability. Note, that the results of the three focal points in Fig. 3, which are separated with a dashed line, were calculated independently. They are combined in one image to illustrate the difference in the focusing capability for different points depending on their position.

**Figure 4:**
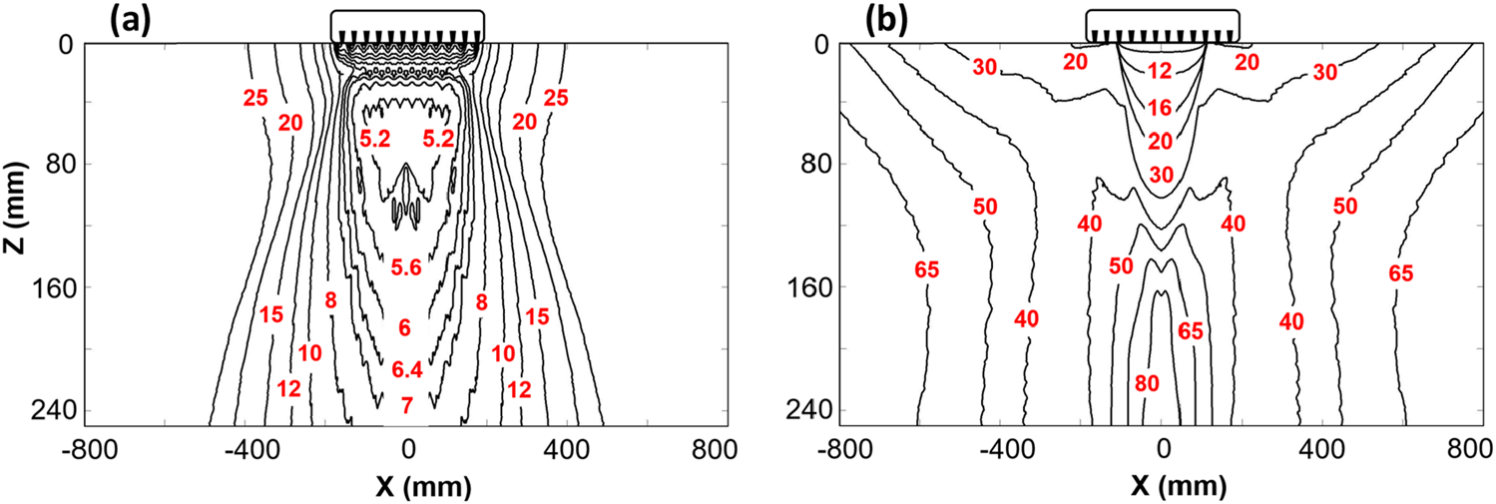
Size of the area (× 100 mm^2^) with a maximum phase shift of 25 mm (Δ*Sp* = 25 mm) around each point in the image plane, using the techniques (**a**) SAFT/TFM and (**b**) HSTFM.

The study is extended to examine the focusing capability of all points in the entire ROI, by calculating the area surrounding the lines connecting  points with a phase-shift of λ/2 = 25 mm. The areas were calculated as per procedure for Fig. 3. The size of areas were estimated by counting of all pixels under the threshold ≤ 25 mm, and multiplication of this quantity by the size of a single pixel (in our case 2 mm × 2 mm = 4 mm^2^). The distribution of the calculated λ/2-area values surrounding each point following the TFM and HSTFM techniques is shown in Fig. 4a, b. It is evident that the areas surrounded by the λ/2-isolines are generally larger in the case of HSTFM in comparison with TFM, which means that the focusing capability of HSTFM is lower than that of TFM. For the case examined here, the best TFM focusing is reached below the array on the acoustic axis at a depth between approximately 40 and 120 mm. It decreases with increasing depth and with increasing lateral displacement from the acoustic axis. For HSTFM we also see minimal values of the 25 mm isoline areas on the acoustic axis below the array. However, as will be explained below, this region of best focus cannot be exploited due to back wall artifacts. The best HSTFM focusing is obtained in a second minimum to the left and the right side of the array, around the 30 and 40 mm isolines. In general, the focusing capability decreases the further the scatterer is from the array.

It has to be noted that such focusing capability estimation is based on the analysis of the phase differences exclusively. In practice, a width of an ultrasonic pulse, structural and electrical noise, and other issues would additionally influence this effect and reduce the focusing quality.

To highlight one more feature of HSTFM, we analyze the points *q* (see Fig. 1), which lie exactly on the line between the imaginary transmitter T* and receiver *R* or between *T* and R*. The summarized Half-Skip propagation path for such a point is constant and equal to the distance between T* and *R* (or *T* and R*), which is denoted in Eq. (5).

5 $${\sum } S=2\sqrt{{d}^{2}+\frac{{a}^{2}}{4}}$$

Here *d* is the thickness and *a* is the distance between transmitter *T* and receiver *R*. This Half-Skip sound path is equal for all the points *q* with coordinates *x*_*q*_ and *z*_*y*_, for which the locus is illustrated in Eq. (6).

6 $${x}_{q}={x}_{r}-\frac{{z}_{q}a}{2d}$$

The half-skip propagation time $t=\frac{2\sqrt{{d}^{2}+\frac{{a}^{2}}{4}}}{V}$ for these points is equal to the propagation time of the wave, which directly propagates from transmitter *T* to receiver *R* via reflection on the back wall surface. This is the ordinary back wall echo. In an object with parallel faces, this signal has a high intensity. In terms of HSTFM reconstruction that means, that all pixels *q* along the line between T* and *R* would have high intensity regardless if there are defects or not. The useful information about any Half-Skip scattering happening in the pixel *q* will be hidden by the strong direct reflection from the back wall.

## 2D simulation of the SH waves in modeled specimens with notches and cracks

**Figure 5:**
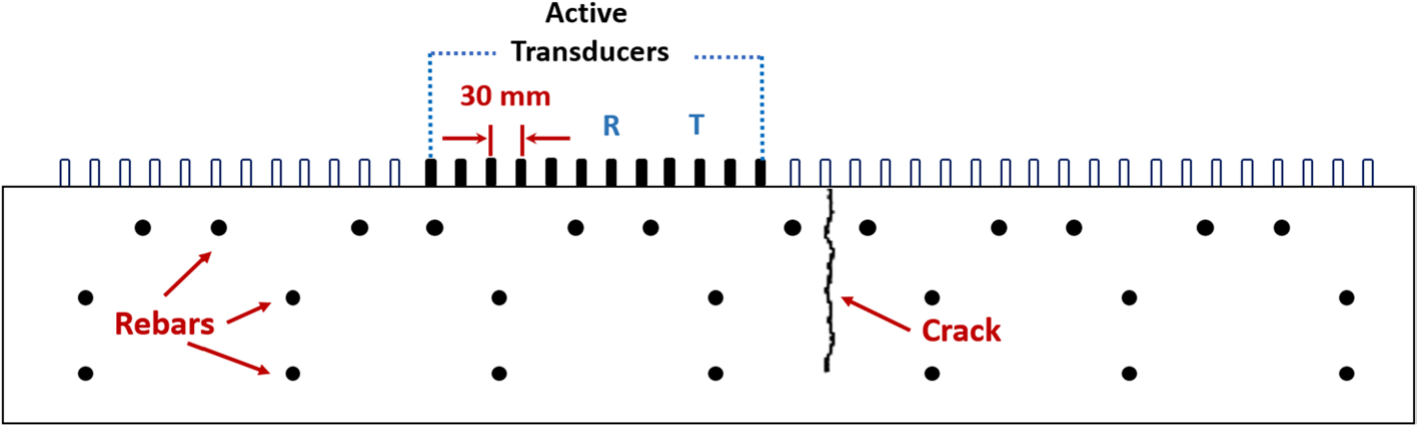
Modeled concrete medium with steel reinforcements and vertical defect.

To investigate ultrasonic wave scattering in Half-Skip mode, and to test the implemented HSTFM algorithm, a set of simplified two-dimensional simulations were used. The simulations were done using the Elastodynamic Finite Integration technique (EFIT)^44,45^. EFIT is a well-known simulation technique for ultrasonic wave propagation, which works on the Finite Difference in Time Domain (FDTD)^44–47^. The material’s elastic properties were discretized in a range of 1/10 of the wavelength. In the model used here, a phase velocity *V* = 2750 m/s was used. This value lies within the interval of 2500–2800 m/s, a range of values determined in our measurements in various studies on concrete structures. The two-dimensional model (Fig. 5) was matched to the cross-section of 250 mm thickness and 1500 mm length of the concrete blocks described in “HSTFM application on experimental data from reference blocks”.

The upper layer of reinforcements with bars of 12 mm diameter and the middle and lower layers with bars of 8 mm diameter are modeled as circular steel sections of their respective diameters. Though concrete is an inhomogeneous material, the medium as a whole was considered as isotropic and homogeneous. To study the effect of the presence of notches and cracks on wave propagation in this modeled medium, notches, i.e., vertical air pockets of varying depths were integrated. Figure 5 illustrates the concrete model with a crack.

The propagation of the shear waves in concrete was simulated by exciting the medium with a Gaussian pulse with a bandwidth of 100 kHz. The emitting and receiving point sources were arranged at a distance of 30 mm in the X-direction in accordance with the properties of the ACS A1040 MIRA ultrasonic array. This instrument features 12 rows of DPC elements each acting like a point-like source or receiver in the X–Z plane. With a single element pitching the incident pulse, the rest of the transducer elements receive the scattered energy. All transducer pairs are sequentially involved in such pitch-catch transmissions in HMC mode. For an arbitrary transducer *n* acting as a transmitter, the elements indexed between *n* + 1 and 12 act as receivers. Sets of 66 ultrasonic A-scans corresponding to one measurement cycle were selected from the simulated data, arranged, and formatted like measured signals. Multiple subsets of the data corresponding to different X-positions of a real instrument were obtained by shifting the position of the simulated array in steps of 30 mm. As the simulation was carried out in 2D, the point sources located in the X–Z plane correspond to line sources in 3D with infinite length in the Y-direction. Each channel of the A1040 MIRA array consists of four DPC sensors with 25 mm spacing in Y-direction, i.e., the real instrument can be considered to be equipped with line sources of finite length. Therefore, the 2D model and simulation provide a good approximation for the sound fields of the linear DPC array.

Two separate simulated studies were carried out, modeling the presence of a vertical surface-breaking notch or a crack individually in a 250 mm thick concrete block. The X-position of the defect was kept constant, and the size of the vertical defects was varied. For each model, the ultrasonic wave fields were simulated and the 66 A-scans were extracted as described above. Table 1 summarizes the model and defect details and other parameters used for the 2D EFIT simulations.

**Table 1:** Summary of parameters involved in simulation-based data acquisition.

Parameter	Value
Dimensions of concrete model (length × thickness)	1500 mm × 250 mm
Size of vertical notches	83 mm, 135 mm and 187 mm
Size of vertical cracks	83 mm, 135 mm and 187 mm
Pulse shape and bandwidth	Gaussian pulse, 100 kHz
Shear wave velocity in model	2750 m/s
Number of transducer channels	12
Inter-element pitch	30 mm
Horizontal position of defects	+ 830 mm in X-direction

## Application of half-skip TFM to simulated data

For full-width ROI reconstruction with the TFM, all scans along the X-direction in a single line were compounded during reconstruction. Compounding means that the data from several positions of the array transducers are superimposed. If these data add coherently, such compounding increases the SNR. This is well known for SAFT/TFM.

Although the HSTFM algorithm can be applied to any scan position of the array, we did not observe such an increase in SNR by compounding. Therefore, the HSTFM images were reconstructed from a single instrument position. The resolution capability of HSTFM follows a trend of the contours presented in Fig. 4. For a reasonable focusing power, a position of the array transducers was selected, in which the distance of the array center from the crack was equal to the thickness of the specimen (250 mm). For this reason, the specimens were examined at a fixed X-position which is 250 mm away from the respective defect location, while the position in Y-direction was scanned.

**Figure 6:**
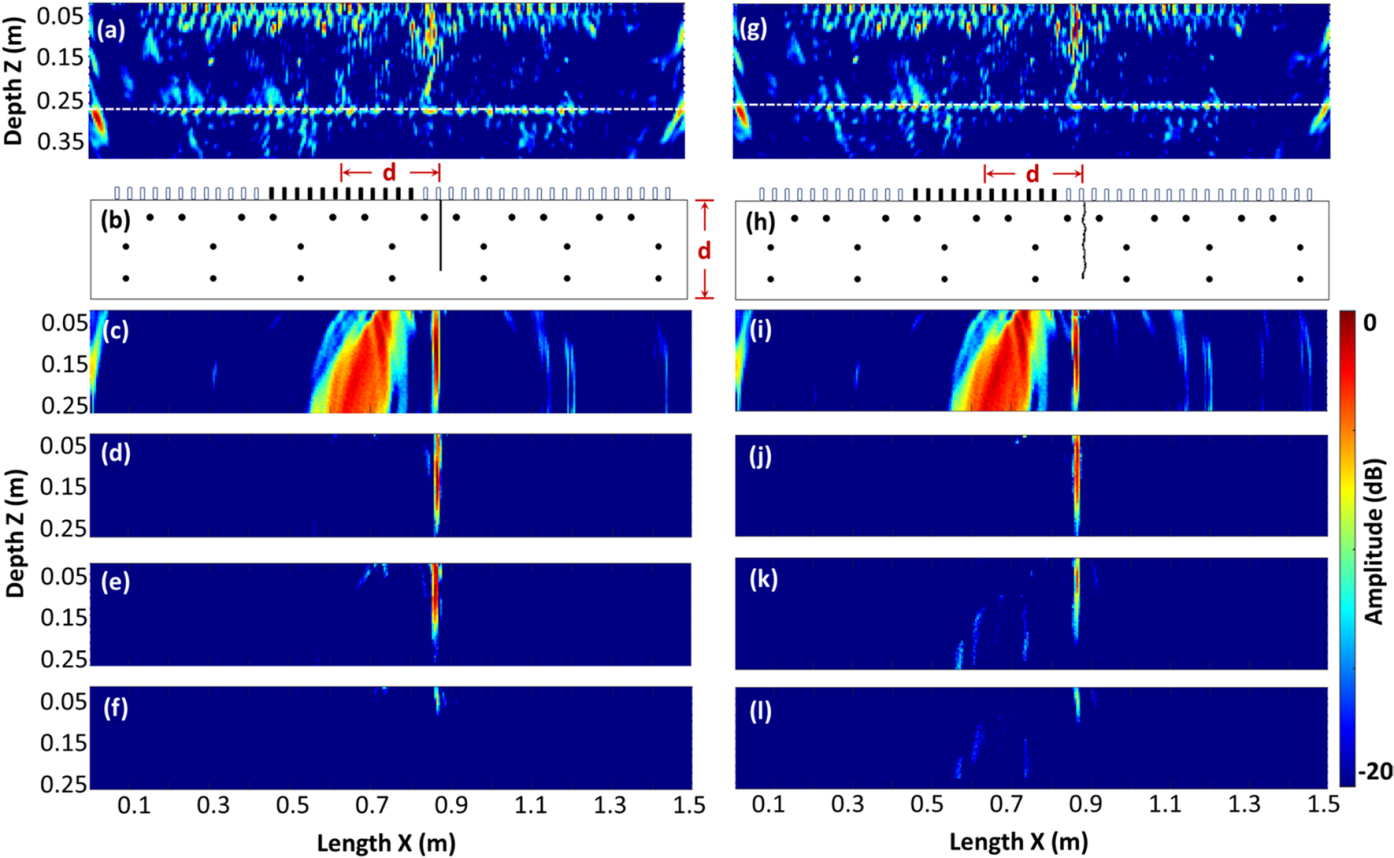
An overview of reconstructed images of the simulated notch and cracks in the concrete model, using (**a**) TFM image for a notch of size 187 mm. (**b**) Positioning of the active array transducers at a distance equivalent to the depth of the specimen, with respect to the notch location. (**c**) Notch reconstructed using HSTFM without time-of-flight filtering. Filtered HSTFM images of simulated data notches of size (**d**) 187 mm, (**e**) 135 mm, (**f**) 83 mm. (**g**) TFM image of the simulated specimen with 187 mm long crack, (**h**) location of simulated array from the crack, (**i**) HSTFM image of 187 mm long crack. Filtered HSTFM images of cracks of size (**j**) 187 mm, (**k**) 135 mm, and (**l**) 83 mm.

Figure 6 shows SAFT/TFM reconstructions of the simulated data. The TFM images (Fig. 6a) have been reconstructed, compounding the data from a linear scan of a virtual array with 12 elements along the X-direction with a step width of 30 mm. Signatures corresponding to rebars in three different layers, a notch at *x* = 830 mm and reflections at the corners of the rectangular model can be visualized with the TFM reconstruction. The boundaries of the modeled concrete specimen are outlined with white dotted lines. The signatures clearly indicate the existence and position of the rebars and the notch in the model. Figure 6c presents the reconstruction results obtained with Half-Skip modes. The image was obtained by programming Eqs. (3) and (4) in MATLAB^48^ and applying the algorithm to data from a single capture (one set of 66 A-scans). The virtual array is laterally positioned away from the defect at a distance equivalent to the thickness/depth of the model of 250 mm as indicated schematically in Fig. 6b, h. A strong signature corresponding to the notch appears at *x* = 830 mm. To the left side of the notch indication, a thick, inclined signature is observed under the position of the array. This undesired, strong signature is a result of the aggregated finite bands of reflections from the specimen back wall (i.e., it is a back wall artifact). Similar patterns in HSTFM images have also been reported in previous research^39,40,49^ on FMC datasets from metallic components. The inclined pattern of the signature observed here is due to the Half Matrix Capture of the array transducers, where all elements transmit individually but only those elements, which did not emit in the previous transmission act as receivers.

To improve the defect visibility, the back wall artifact was removed from the reconstruction process by integrating a minimum time-of-flight into the reconstruction algorithm. Only that part of the A-scans with times-of-flight higher than the two-way travel time to the specimen bottom (Eq. 5) was considered for imaging (Fig. 6c). This approach significantly reduces the dominance of the back wall reflection artifact. As all images were normalized to their maximum amplitude the visibility of the defect signature is improved. Such strategies to suppress the strong artifacts which are caused by the direct back wall reflections in Half-Skip images have also been reported earlier^49^.

To benchmark the implemented HSTFM imaging technique for concrete, its performance was tested with the notches of different depths within the simulated data as listed in Table 1. The clearly pronounced vertical indication of the crack or the notch (*x* = 830 mm) is present in all cases in Fig. 6. Results (Fig. 6d–f) show corresponding changes in the size of the defect patterns with varying notch sizes. Note that because of the special properties of the HSTFM algorithm, other features such as rebars or the back wall are not visible in these reconstructions. The captured Half-Skip signals from the rebars have a significantly lower intensity than the signature of the crack or notch. A reinforcement bar acts like a single-point scatterer in the image plane, because the diameter of the rebar (8 mm or 12 mm) is smaller than the wavelength of the ultrasound (50 mm). This point sources scatter the acoustic energy in all directions. On the other hand, the crack and notch are extended vertical reflectors. Indications of such reflectors were specifically enhanced in the HSTFM image by choosing one special position of the instrument aperture and superimposing the scattered waves from different points of these vertical reflectors. Since the back wall and reinforcement are very well displayed in the standard TFM mode, and this work aims to evaluate vertical cracks, the dynamic range of the color table is chosen so that the vertical cracks are well visible. Whether reinforcement bars can also be mapped by HSTFM was not the subject of this work but could be investigated in the future.

**Figure 7:**
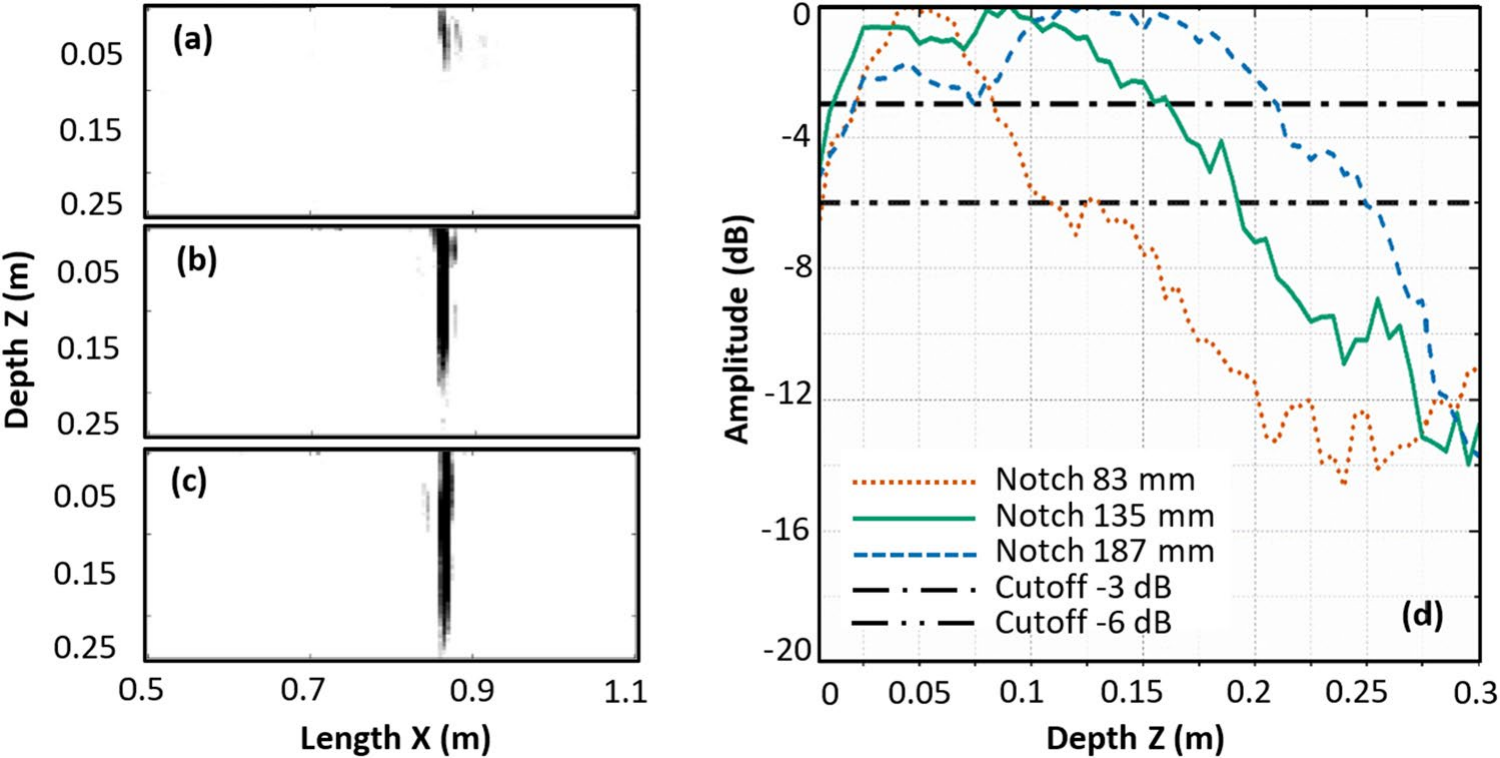
Binary image of pixels corresponding to defect signatures thresholded at −6 dB amplitude level in the ultrasonic HSTFM images of simulated notches of size (**a**) 83 mm, (**b**) 135 mm, (**c**) 187 mm. (**d**) Amplitude–depth plots for defect signatures of all three notches.

With changing sizes of the Half-Skip reconstruction signatures of the cracks and notches being evident, an approximate defect sizing methodology has been adopted using the −3 dB^50^ and −6 dB amplitude drop methods^39^. The binary image (threshold set at the amplitude level of −6 dB) in Fig. 7a–c indicates the pixels exclusively corresponding to the defect signature in a selected sub-region of the HSTFM image of the notches sized 187, 135 and 83 mm (Fig. 6d–f). The maximum amplitude of all pixels in each horizontal row of the defect signature was plotted to study the amplitude-depth trends. Figure 7d plots the amplitude variation of the defect signature along the depth of the reconstruction with respect to its maximum amplitude obtained in this way. To define a defect depth from these plots, thresholds were set at −6 dB and −3 dB with respect to the maximum amplitude. The depth values at these thresholds are regarded as probable defect sizes. The estimated depths using threshold values are listed in Table 2.

**Table 2:** Comparison of actual defect sizes with the estimated defect sizes using the −6 dB and −3 dB drop methods.

Defect type (simulated)	Actual size of defect (mm)	Measured size using −6 dB drop (mm)	Error (%) with −6 dB drop (%)	Measured size using −3 dB drop (mm)	Error (%) with −3 dB drop (%)
Notch	83	100	+ 20.48	80	−3.61
Notch	135	185	+ 37.04	150	+ 11.11
Notch	187	245	+ 31.01	200	+ 6.95
Crack	83	115	+ 38.55	90	+ 8.4
Crack	135	190	+ 40.74	125	−7.4
Crack	187	235	+ 25.66	190	+ 1.6

The results of a corresponding evaluation using the models with simulated cracks with different depths (Table 1) in the specimen (Fig. 6i–k) confirm the applicability of the HSTFM technique for vertical crack detection.

**Figure 8:**
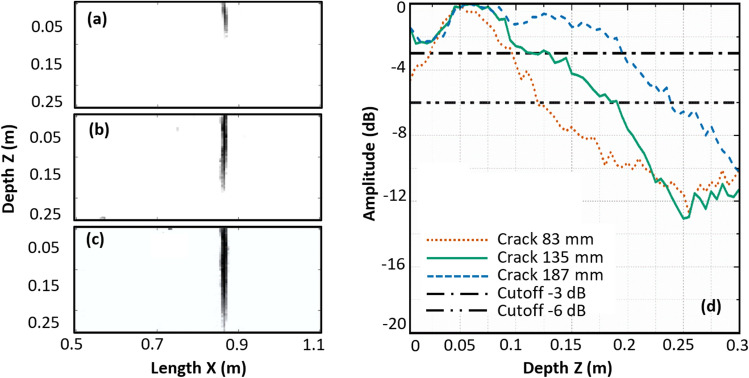
Binary image of pixels corresponding to defect signatures thresholded at −6 dB amplitude level in the ultrasonic HSTFM images of simulated cracks of size (**a**) 83 mm, (**b**) 135 mm, (**c**) 187 mm. (**d**) Amplitude–depth plots for defect signatures of all three cracks.

Repeating the reconstruction for the different crack sizes in the simulated specimens also shows a changing pattern of signatures with crack size (Fig. 6j–l). The same defect sizing strategy as used for the notches has been applied on pixels from the generated binary image (Fig. 8a–c). Amplitude-depth plots of crack signatures are shown in Fig. 8d. Table 2 summarizes the depth estimated from the HSTFM images using the −6 dB and −3 dB drop methods and the actual depth of the cracks as implemented in the models.

The amplitude thresholds of −3 dB and −6 dB (with respect to the maximum intensity of the defect signature) were chosen by the following considerations: Felice^39^ used the −6 dB drop for the crack sizing by HSTFM in metals. Table 2 shows however, that for the concrete specimens and defects, examined in this study, the −3 dB amplitude threshold delivers more precise results.

Typical threshold values like −6 dB, −10 dB, −20 dB have been used in ultrasonic inspection for many years. These thresholds are in close relationship with the sound field of a single transducer, which is commonly used in conventional ultrasonic inspection. The interpretation of the results of conventional ultrasonic inspection is based on the analysis of the reflected sound field. The principles of the image creation and its analysis in tomographic methods differ from the conventional ultrasonic inspection. Thresholds in SAFT/TFM are usually set based on empirical experience. To make sure that the −3 dB threshold value provides optimal results, we analyzed the relationship between the threshold values in a range between −0.5 and −7 dB and the evaluated crack depth for the HSTFM results of simulated data.

**Figure 9:**
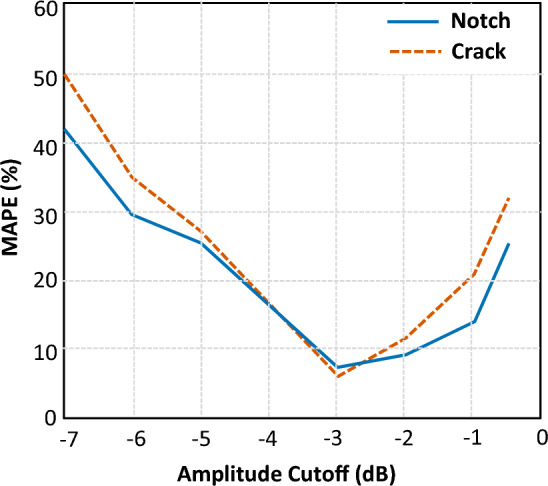
Mean Absolute Percentage Error of the defect depth estimation as a function of used amplitude threshold, HSTFM of simulated data.

Figure 9 shows the Mean Absolute Percentage Error (MAPE) of the notch/crack depth estimation as a function of the threshold level:

7 $$MAPE=\frac{100\%}{m} \sum \left|\frac{{d}_{e}-{d}_{a}}{{d}_{a}}\right|$$

Here *d*_*e*_ is the depth, evaluated from the HSTFM reconstruction, *d*_*a*_ is the actual vertical size of the defect, which was a parameter of the simulation model. The error values were averaged for *m* = 3 simulated datasets (defect depths 83, 135 and 187 mm).

It becomes clear that −3 dB is the most appropriate level to be set as the amplitude cutoff. Note that this is an empirical finding for the case simulated here.

**Figure 10:**
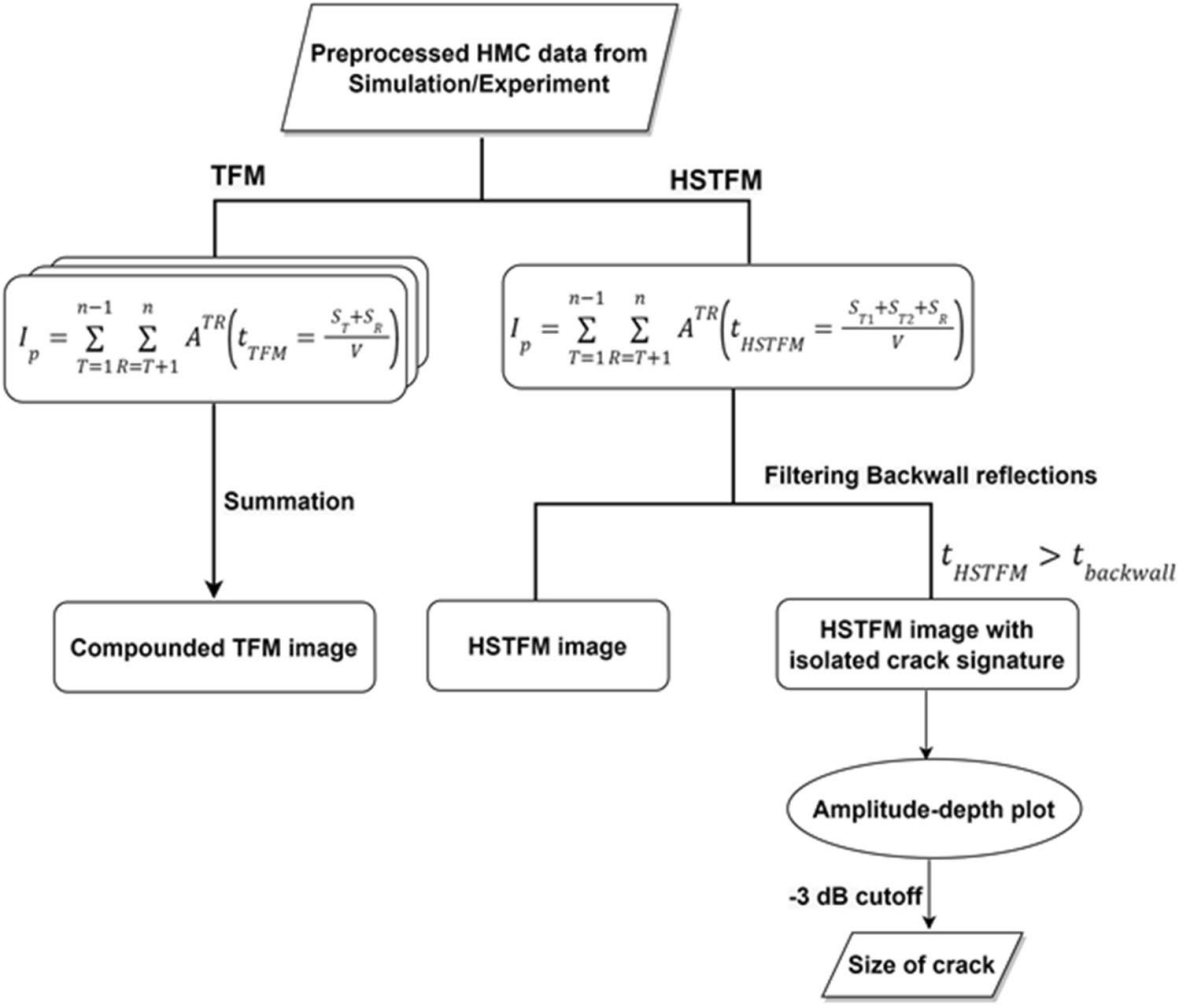
Flowchart showing steps involved in reconstruction using TFM and HSTFM.

From the studies of HSTFM reconstruction using simulated data, it can be concluded that the Half-Skip signatures of both notch and crack are clearly identifiable, continuous, as well as dependent on defect size. All these factors together, favor the back calculation of defect sizes to arrive at a reliable estimate. It appears that the sizes estimated using the −3 dB amplitude drop method are closer to the actual depths when compared to those obtained from the other cutoff thresholds (Fig. 9). The entire process of imaging and sizing cracks adapted through this simulation study is demonstrated as a flowchart in Fig. 10.

## HSTFM application on experimental data from reference blocks

### Specimen preparation

For experimental validation of the proposed HSTFM, four Reinforced Concrete (RC) specimens with identical dimensions (1500 mm × 600 mm × 250 mm) have been used. They will be addressed as Specimens S1, S2, S3 and S4 in the succeeding sections of this article. The specimens were fabricated in accordance with the procedure described in^43^ to create natural cracks of limited depth, which are stable over time.

The concrete has been mixed for BS grade C30/37 and the reinforcing bars used are of diameters 12 mm (top layer) and 8 mm (middle and bottom layers). The upper and lower layer of reinforcement are identical for all specimens. The depth of the middle layer is tailored to the type of defect planned with each specimen. Table 3 presents details of the specimens with their types of defects. Specimen S1 was planned to be grooved by cutting with a saw until a depth of 75 mm to create a vertical notch. Thus, reinforcements were lined up at the depth of 85 mm to prevent the concrete from cracking beneath the notch. Similarly, the middle layer of reinforcements is adjusted for other specimens to generate different crack depths^43^. After casting, the specimens were left for curing until 28 days.

**Table 3:** Summary of planned defect locations in experimental specimens.

Specimen no.	Planned defect	Defect location along X-direction (mm)	Concrete cover of rebar layers (Z-coordinate)		
			Top (mm)	Middle (mm)	Bottom (mm)
S1	Notch, 75 mm depth	1070	40	85	210
S2	Partial-depth crack, constant depth	850	40	125	210
S3	Partial-depth crack, constant depth	850	40	165	210
S4	Partial-depth crack, variable depth	1100	40	125	210

### Creation of controlled cracks and notch

**Figure 11:**
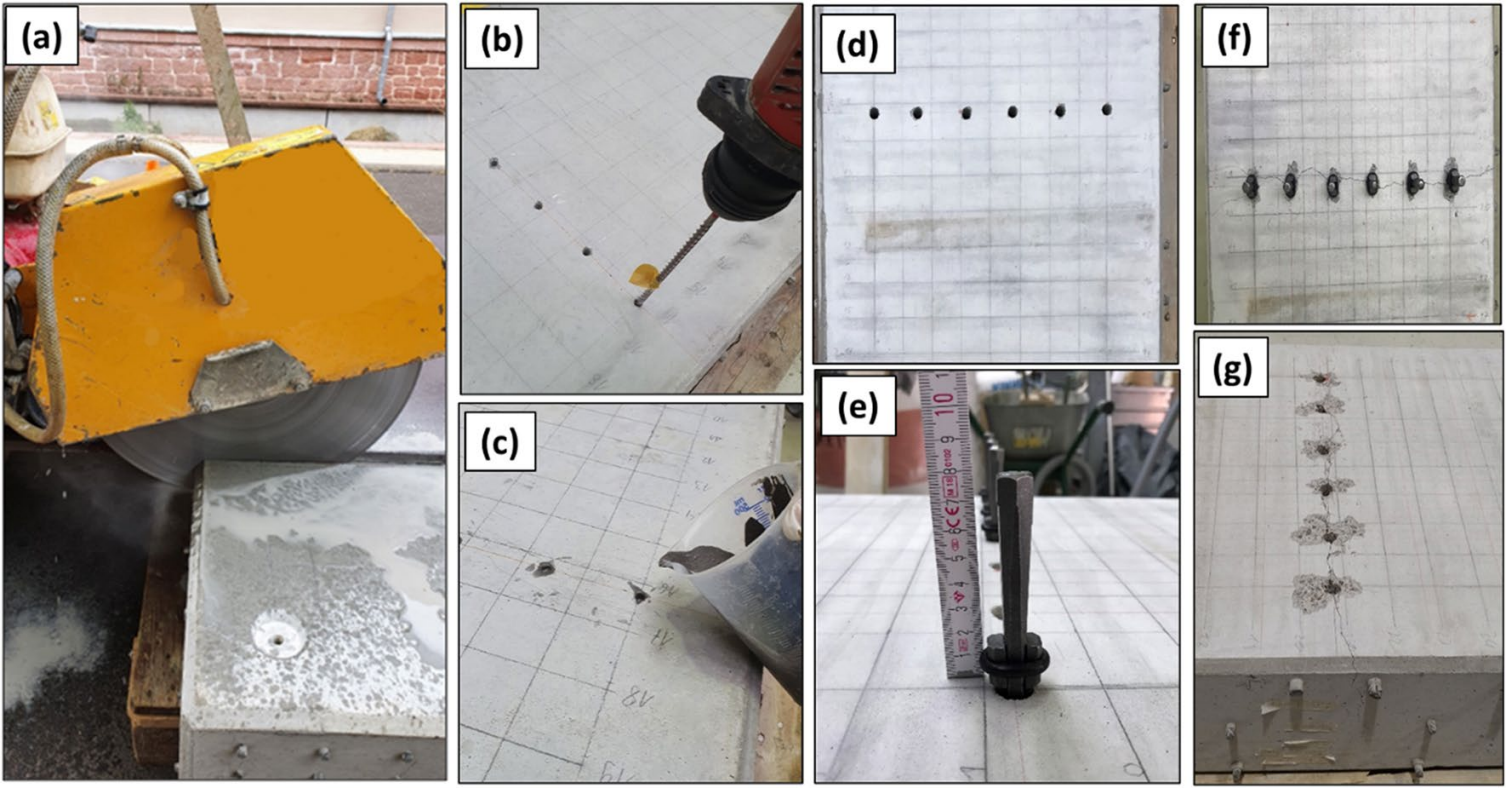
(**a**) Generating a groove in Specimen S1 using a joint cutter blade, (**b**) drilled boreholes for Specimens S2 and S3, (**c**) injecting expansive Bentonamit mortar into boreholes, (**d**) boreholes for cracking in Specimen S4, (**e**) hammered wedges into the boreholes for cracking the specimen, (**f**) intermediate stage of cracking, (**g**) stable crack after all stages of cracking.

To generate a controlled saw-cut in Specimen S1, a joint cutter was used as shown in Fig. 11a. The specimen was grooved up to a depth of 75 mm from the surface to such that a vertical notch was formed.

The cracking of specimens S2 and S3 was done by injecting expansive mortar (Betonamit) into a row of drilled boreholes (Fig. 11b, c) at the top surface, as devised in^43^. Betonamit is a swelling clay material that develops pressure with reaction time. Usually cracks open up within 72 h after injecting the expansive mortar. The interior reinforcements—especially the middle layer—counteract to limit the crack growth into deeper parts of the specimen. In order to create cracks with different depths the middle layer of rebars was placed at different depth positions in specimens S2 and S3.

For specimen S4, controlled stage wise cracking was performed. On the top surface of the healthy specimen, a row of six drill holes was created with 65 mm depth and 10 mm diameter (Fig. 11d). The positions of the drills were planned to avoid damaging the top layer of rebars. Tapered metal splitting wedges were inserted into the drill holes and used for stage wise cracking of the specimen from the surface. One wedge was plugged into each drilled borehole separately and each wedge was repeatedly struck by a hammer on its center (Fig. 11e). All wedges have been ensured to penetrate for the same depth during each cracking stage (Fig. 11f). After the surface was cracked, one wedge was removed to make space for the MIRA A1040 instrument, and an ultrasonic line scan covering the cracked region was recorded. Subsequently, the remaining wedges were inserted deeper into the drill holes with additional hammer blows. After each stage of row-by-row impact loading, ultrasonic inspection was carried out by scanning the identical lines. In addition, the width and the depth of the visible crack at the surface and at the side faces were recorded at every stage. The procedure was continued until the visible crack at the side faces was near to the depth of 200 mm (Fig. 11g).

**Figure 12:**
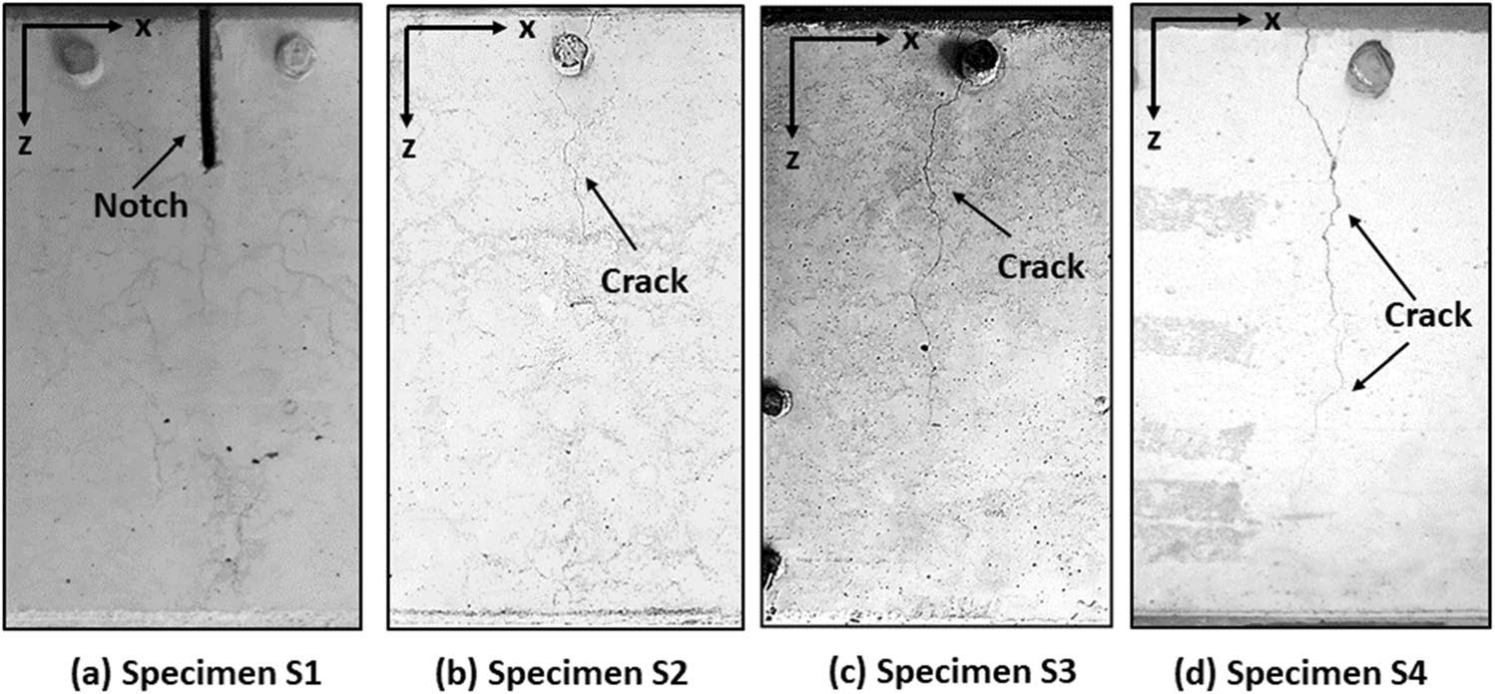
Generated cracks viewed from the side faces of (**a**) Specimen S1 with the notch, (**b**) Specimen S2 with crack, (**c**) Specimen S3 with crack and (**d**) Specimen S4 with crack after final cracking stage.

Side views of all four specimens that show the defects, at their stable final stages, are presented in Fig. 12.

### Data collection on reference blocks using the ultrasonic array

**Figure 13:**
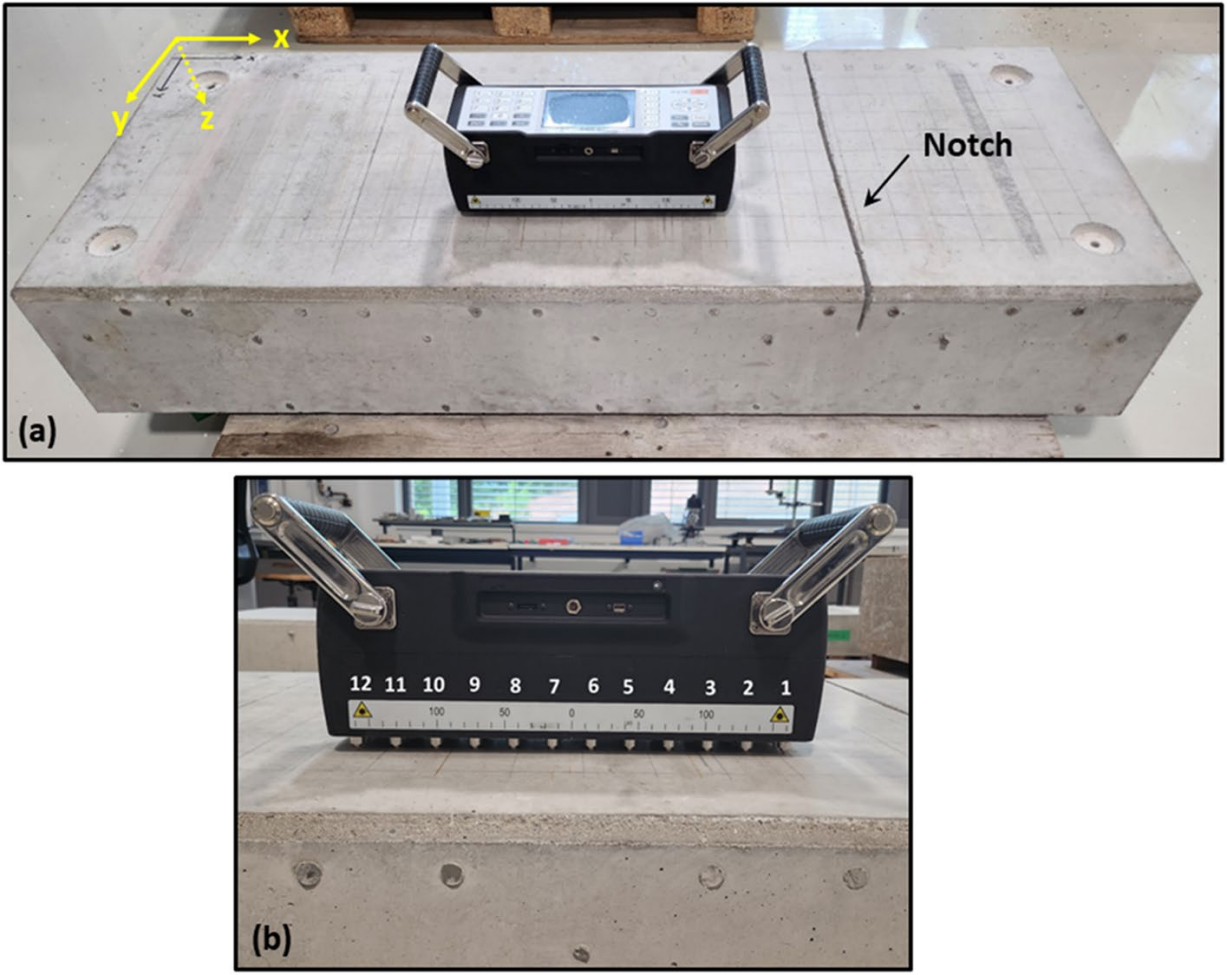
(**a**) Setup showing inspection of the specimen with ultrasonic array (ACS A1040 MIRA) in position *x* = 740 mm, *y* = 400 mm, (**b**) indexing of transducer channels for sequential pulse excitement.

The ultrasonic inspection was carried out with a 12-channel shear wave DPC transducer array (ACS A1040 MIRA). The nominal center frequency of the excited ultrasonic pulse was 50 kHz. A typical inspection setup is shown in Fig. 13a. As previously explained, this system operates with the HMC principle. The rightmost channel indexed as transmitter #1 is first operated in each recording cycle, as illustrated in Fig. 13b.

Every channel of the linear array is a combination of four spring-loaded, point-like DPC transducers, each separated by 25 mm in the direction perpendicular to the instrument axis. The direction of vibration of the transmitted shear wave is perpendicular to the instrument axis (SH orientation). The pitch of the 12 rows in the instrument axis (active aperture) is 30 mm. A single measurement of the array sequentially excites shear pulses from the channels indexed 1—11, as illustrated in Fig. 13b. The emitted pulses get scattered and are captured by the receiving transducers indexed with higher numbers than the transmitting elements (HMC). This results in a collection of 66 scattered time signals. The 66 A-scans are recorded with a signal length of 2048 μs and a sampling frequency of 1 MHz.

All measurements consisted either of manually taken line scans or area scans on the X–Y surface of the specimen recorded using the “map mode” of the MIRA instrument. The geometric center of the array system is considered as the positional reference. The area scans consisted of nine lines with a spacing of 50 mm in the Y-direction between the lines. Each line is scanned from *x* = 200 mm to *x* = 1280 mm with a step width of 90 mm or 50 mm along the X-direction. Specimens S1, S2, and S3 feature defects of stable depth. Several surface scans were taken on each specimen. A comparison of the ultrasonic data showed slight variations in signal amplitude probably due to a limited precision of the manual positioning of the instrument. However, the main features (echoes) in the signals and in the reconstructed images (indications) were reproducible in their position and shape. Specimen S4 was inspected once after each cracking stage. The extracted pulse-echo data *A*^*TR*^ in time-domain were corrected for DC bias and then used for either TFM or HSTFM reconstruction.

The sound velocity in each specimen was measured based on the position of the back wall indication in the TFM reconstruction, and on the comparison with the actual thickness of the specimen. For all specimens (S1–S4) the sound velocity was 2530 ± 10 m/s. In accordance with the procedure described in chapter 4, for full-width reconstruction with TFM, all scans along the X-direction in a single line were compounded during reconstruction. The HSTFM images presented in the following figures were calculated from one single instrument position. To obtain a reasonable focusing power, a position of the array was selected, in which the distance of the array center from the crack was equal to the thickness of the specimen (250 mm), i.e., the specimens were scanned across the Y-direction at a single X-position which is 250 mm away from the respective defect location.

## Results and discussion

Experimental data from all the specimens (S1–S4) was post-processed using the TFM and HSTFM techniques. Amplitude data from the reconstructed HSTFM images were extracted to plot amplitude-depth plots for estimating the depth of crack penetration. The performance of the HSTFM was experimentally evaluated on specimens with stable and varying crack depths.

### Evaluation of specimens with stable crack depths

**Figure 14:**
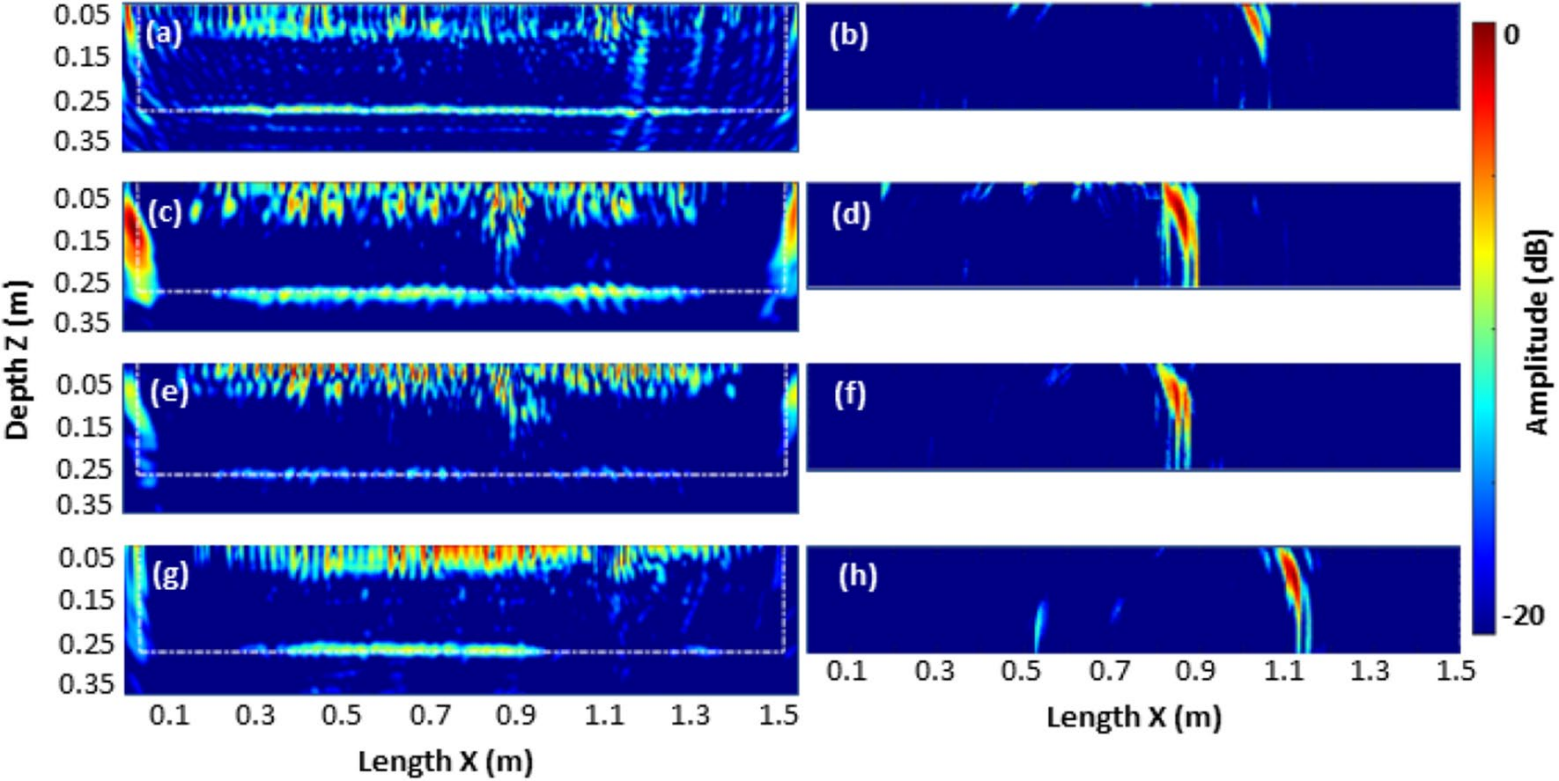
Comparison of the ultrasonic images of specimens S1–S4, reconstructed using the TFM and HSTFM techniques. Specimen S1 imaged using (**a**) TFM, (**b**) HSTFM, S2 with (**c**) TFM, (**d**) HSTFM. S3 reconstructed with (**e**) TFM. (**f**) HSTFM and S4 with (**g**) TFM and (**h**) HSTFM.

Figure 14 compares the imaging results in SAFT/TFM and HSTFM modes of all specimens S1–S4 with stable defects (notch and cracks). The reconstructed TFM images of all specimens are presented in Fig. 14a, c, e, g. White dotted lines overlaid on the ultrasonic TFM images indicate the actual boundaries of the specimen. The TFM images commonly indicate the reflections from the flat specimen back wall at *z* = 0.25 m and layers of rebars in between. Although the presence of reinforcements in multiple layers is evident, their intensities are low in comparison to that of the back wall. Signatures corresponding to the cracks in the TFM images (at *x* = 1.07 m for S1, *x* = 0.85 m for S2 and S3, and at *x* = 1.1 m for S4) are accompanied by shadowing of the bottom reflection signatures below the crack. On the other hand, the HSTFM-based images reconstructed from the experimental data are shown in Fig. 14b, d, f, h. The images reconstructed are from scan positions *x* = 0.83 m for S1, *x* = 0.6 m for S2 and S3, *x* = 0.85 m for S4. As demonstrated in "Application of half-skip TFM to simulated data", the HSTFM images come with strong direct back wall reflections under the position of the array, which appears to be a dominant artifact with respect to the defect signatures. The presented images are calculated with suppressed bottom reflections following the filtered algorithm that only considers travel times greater than that of the back wall. The images show a clear and continuous signature corresponding to the notch/cracks present at respective locations in specimens S1–S4.

While the TFM reconstruction indicates the position and nature of the crack indirectly, by shadowing the backside echo in the reconstruction or through the corner echo when reconstructed within the limited open angles for a full-depth crack^26^, the HSTFM technique maps the crack directly.

**Figure 15:**
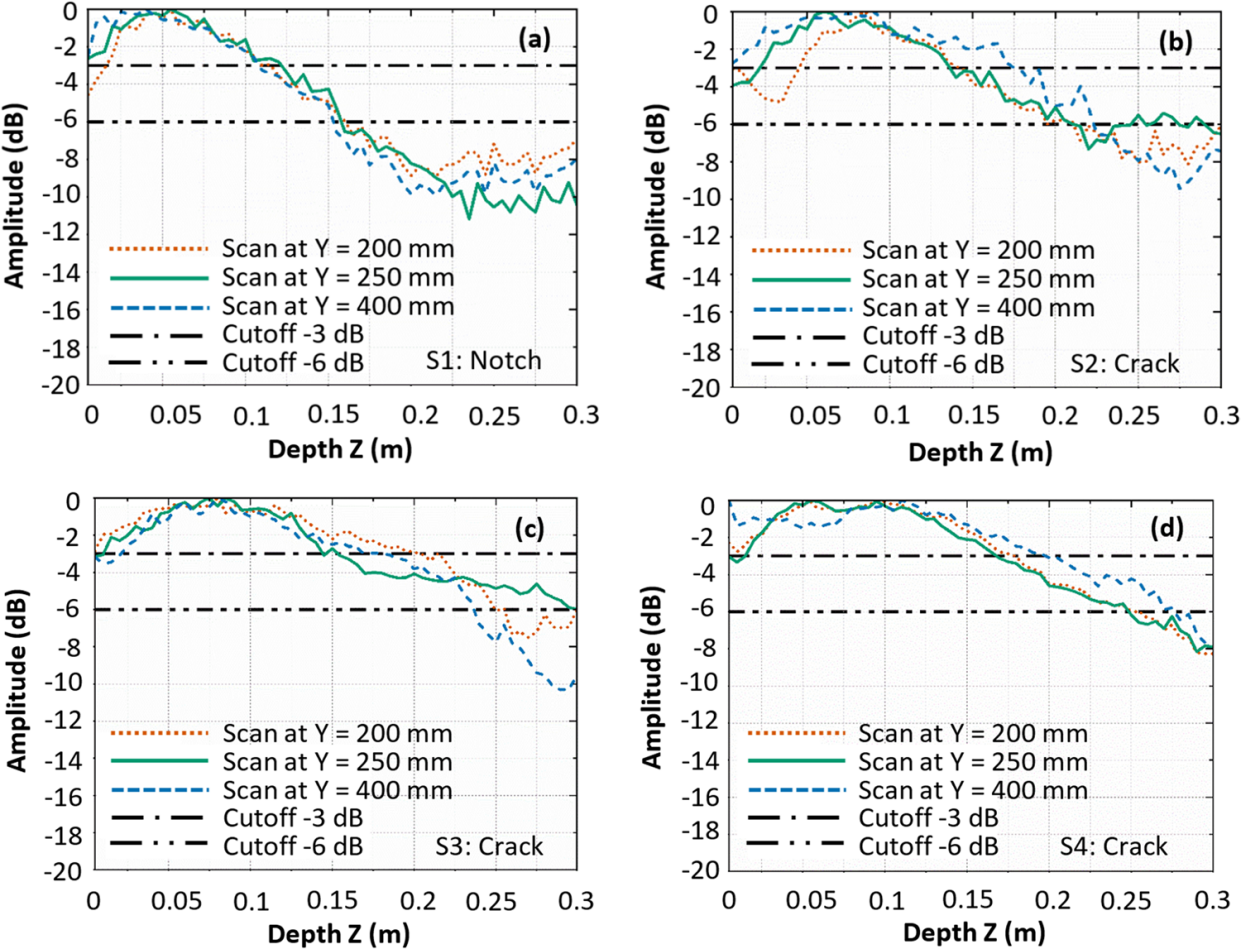
Amplitude-depth plots derived from the HSTFM images of (**a**) Specimen S1, (**b**) S2, (**c**) S3 and (**d**) at the final stage of specimen S4. The three different amplitude profiles in each figure were obtained from three different scan lines taken with the same X-values for each specimen but different Y-values (see coordinate system shown in Fig. 13).

To quantify the depth of cracks, the amplitude-depth plots along the ultrasonic signature in the HSTFM images are plotted in Fig. 15, following the procedure explained in chapter 4 (for Figs. 7 and 8). To ensure the reproducibility and the statistical accuracy of the results, three independent measurements were taken along the Y-direction, with identical distances in X-direction to the crack/notch in each specimen. The notch in specimen S1 is an artificial defect representing a crack of uniform depth across its width. The HSTFM data obtained from this specimen are used to calibrate the threshold for the depth estimate in the experimental setup. As the depth of the notch is constant in width (Y) direction, the three different measurements at different positions Y (see Y-values in Fig. 13) should provide the same depth value. As seen in Fig. 15a, the −6 dB threshold overestimates the depth of the notch to 140 mm, while the value at −3 dB (80 mm) is in excellent agreement with the experimental situation. This finding confirms the results obtained with the simulated data (Table 3), where the −3 dB threshold also provided a better estimate for crack depth.

Following the same strategy with a −3 dB cutoff for depth estimation, specimens S2 and S3 exhibit an average measured depth of 145 mm and 170 mm respectively from Fig. 15b, c. The measured depth of crack in Specimen S4 at its final stage of cracking is 180 mm as derived from Fig. 15d. The actual crack depth cannot be determined non-destructively, but^43^ reports a uniform crack shape when following the procedure outlined therein. The estimated crack depths indicate the extent of the crack propagation with an expected error rate summarized from the simulations. Table 4 presents a comparison between the actual depth of cracks obtained from the visual inspection and the depths measured from the amplitude-depth plots (−3 dB) of the reconstructed HSTFM images. The visual inspection-based crack depths are measured from the visible crack patterns at the exposed surfaces orthogonal to the crack. Measurements made through visual examination are affected by inaccuracies (± 16 mm) due to the presence of aggregate of maximum size 16 mm. Thus, the actual crack depth in the internal regions of the specimen is expected to be within the limits of the observed range.

**Table 4:** Summary of observed crack depth at side faces of specimens S1, S2, S3 and S4 and respective estimated depths from HSTFM images.

Specimen	Observed defect depth at side faces (mm)	Measured defect depth using −3 dB amplitude threshold (mm)
S1	75 (notch)	80
S2	125 $\pm$ 16	145
S3	165 $\pm$ 16	170
S4	190 $\pm$ 16	180

### Evaluation on varying crack depths

**Figure 16:**
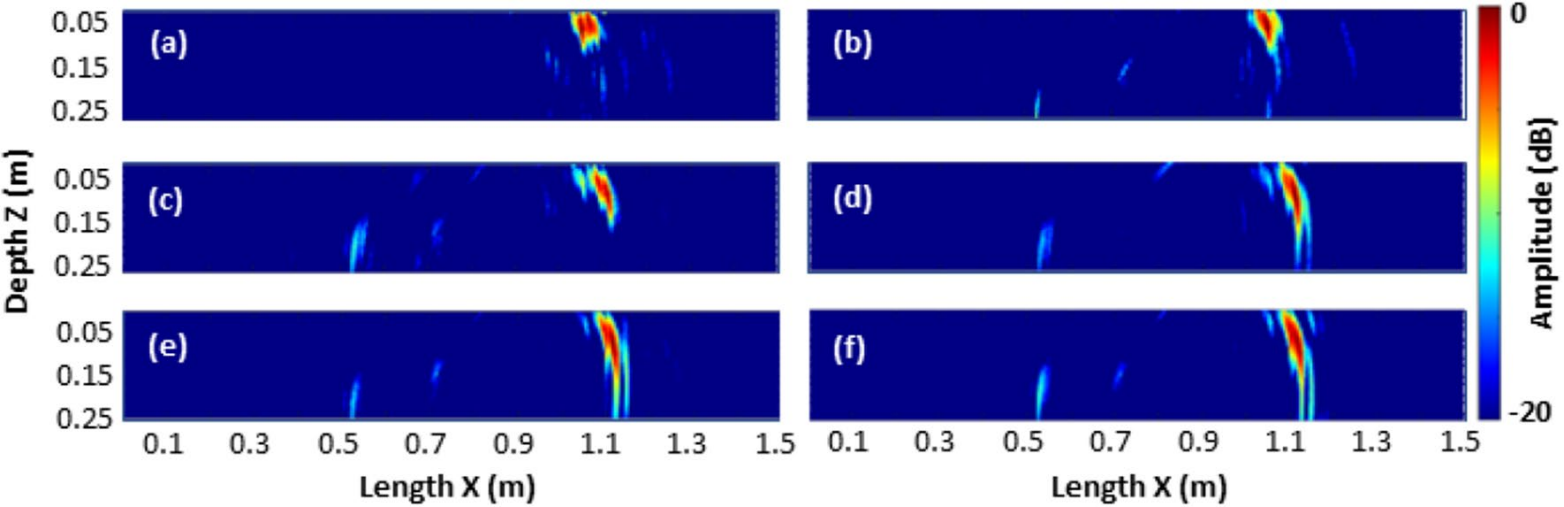
Images of specimen S4 reconstructed with the HSTFM technique at (**a**) drilled stage, (**b**) Stage 1 of controlled cracking, (**c**) Stage 2, (**d**) Stage 3, (**e**) Stage 4, (**f**) Stage 5.

On specimen S4, a set of measurements was taken while the crack depth was increased iteratively. In this special case, data is available for a crack with increasing depth. Images reconstructed with the HSTFM technique at every cracking stage are presented in Fig. 16. They were reconstructed using the data captured at *x* = 850 mm.

As the crack progresses stepwise in depth the signature at *x* = 1.1 m grows correspondingly. The Half-Skip signature extends to the near-bottom of the specimen in the last stage (Fig. 16f), where the visible crack pattern at the exposed edges of the specimen is at 190 ± 16 mm.

**Figure 17:**
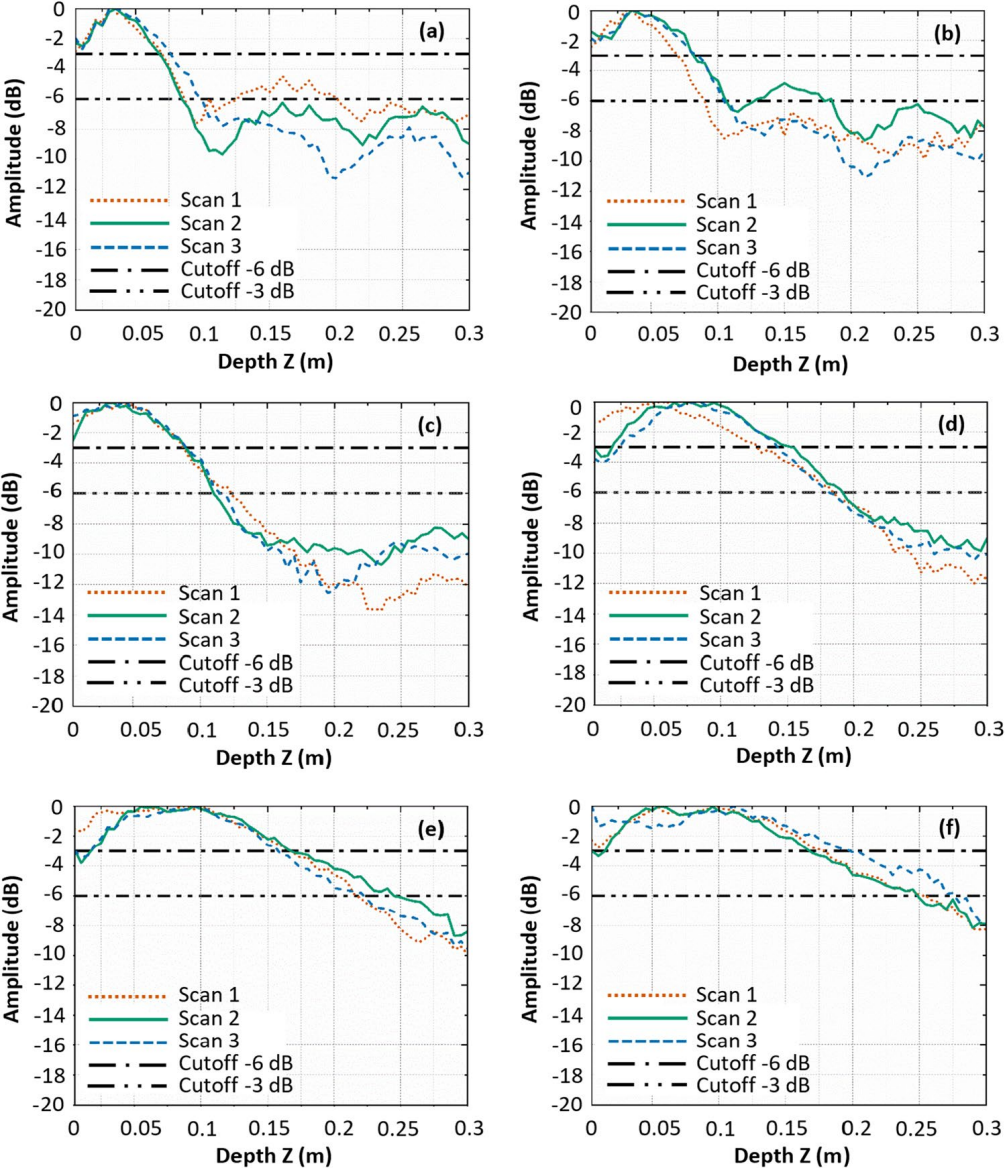
Amplitude-depth plots of defect signatures in reconstructed HSTFM images of specimen S4 at (**a**) drilled stage, (**b**) Stage 1 of controlled cracking, (**c**) Stage 2, (**d**) Stage 3, (**e**) Stage 4 and (**f**) Stage 5.

For each damage level of specimen S4, the amplitude-depth plots (Fig. 17) were determined from the measured ultrasonic data, as described previously. The crack size was estimated with the −3 dB amplitude drop method and averaged over three different measurements after every stage of cracking. In Table 5, the values obtained by HSTFM are listed against the observed depth of crack at the side faces of the specimen. The fact that the measured depth increases as the crack progresses, confirms the depth sensitivity of the HSTFM technique.

**Table 5:** Summary of observed crack depth at side faces of specimen S4 and estimated depths from HSTFM images.

Cracking stage	Observed defect depth at side faces (mm)	Measured defect depth using −3 dB amplitude threshold (mm)
Drill holes	65	72
Stage 1	80 $\pm$ 16	72
Stage 2	120 $\pm$ 16	90
Stage 3	160 $\pm$ 16	140
Stage 4	170 $\pm$ 16	158
Stage 5	190 $\pm$ 16	180

It is observed through several experimental investigations using ultrasonic imaging and after applying the −3 dB drop rule to the HSTFM images that the measured crack depths from the ultrasonic images are in fair agreement with those from visual examinations. Therefore, the HSTFM is found to be suitable for resolving near-vertical discontinuities. However, further studies are still required to quantitatively evaluate natural cracks by ultrasonic imaging under field service conditions.

## Summary and conclusion

This study investigates the application of the half-skip total focusing method for the processing of ultrasonic multi-static data to analyze surface-breaking cracks in concrete. The HSTFM method has been proposed by others for depth measurement of small SBCs in metals^39^ and was recently also applied to artificial planar defects in concrete^30^. The HSTFM mode makes use of signals, which are reflected once at the back wall, before or after they are scattered by the defect. In this work, the HSTFM algorithm was tested for a linear array of DPC transducers, which generate SH waves with the central frequency of 50 kHz. The local resolution of the method was plotted in comparison to the local resolution of standard TFM. In accordance with the work on metals it was shown that, in cases when flat concrete blocks are examined, the area directly below the array is not accessible to half-skip mode because of a strong back wall artifact, which covers any other indication of defects in this area.

The HSTFM reconstruction was first tested on simulated data obtained by 2D modeling using the elastodynamic finite integration technique. The distance of the point sources of the simulation model was 30 mm in accordance with the pitch of the available linear array. The simulated A-scans were also arranged in the same way as the experimental data taken in Half Matric Capture mode with the experimental instrument. By processing simulated data sets of notches and cracks with different depths, it was shown that clear defect signatures can be obtained in the images after the back wall artifact is removed. The reconstruction of numerically generated model data clearly showed that HSTFM—after filtering the direct reflections from the back wall—could localize the position of the cracks/notch and size their depth. From the results, it also appears that the presence of nominal reinforcements has no direct effect on the crack signature. A defect sizing strategy based on a −3 dB drop in the amplitudes of the ultrasonic signature with respect to its maximum has been found to indicate the depth of the crack/notch under investigation. The defect signatures were quantified with this −3 dB drop method to obtain an estimated value of notch or crack depth.

Experimental data obtained on four different specimens with the dimensions 1500 mm × 600 mm × 250 mm (length × width × thickness) were processed. One specimen was with a notch and three specimens with cracks. Clear defect signatures were obtained on all specimens after the back wall artifact was removed from the images. The crack or notch depth was analyzed again with the amplitude drop method as developed for the simulated data. As the crack or notch depth could be considered approximately constant in the Y-direction, measurements with three different Y coordinates but identical X coordinates were compared for each specimen. For the notch for which the depth is known, the − 3 dB drop method provided the closest result for the depth value estimated by the ultrasonic HSTFM method. For the specimens with cracks, the exact crack depth can only be interpolated by using the depth values obtained by optical measurements at the side faces of the specimens. The average deviation of the crack depth values as obtained by the − 3 dB drop method from the values measured optically at the side face was less than 16 mm, i.e., the average error is less than the aggregate size. This error is close to the aggregate size of 16 mm. The depth sensitivity of the HSTFM technique was evaluated using a specimen with a step wise increase in crack depth. The results indicated a growing trend of reflection signatures and estimated sizes using the −3 dB drop method.

The HSTFM results were also compared with the well-known and widely applied SAFT/TFM analysis. SAFT/TFM can reliably localize features parallel to the top surface (back wall, cracks parallel to the surface, delaminations^30^) on which the ultrasonic device is positioned, as well as structural elements like reinforcement and tendons. However, vertical features like cracks do not directly reflect the incoming sound wave back to the receiver and are therefore not directly localized or sized. While reconstructions based on the SAFT/TFM give an indirect indication of the crack position, they do not allow measuring the crack depth. In addition, using TFM, both defect types (notch and crack) were mapped much better in simulation than in the experiment with natural cracks. However, implementing half-skip travel modes to the time-domain reconstruction improved the reflection signatures corresponding to the defects in all cases.

It has been shown that HSTFM-based processing is a valuable addition to the standard SAFT method when surface breaking cracks are to be examined. It has the potential to grow into a standard method to evaluate surface-breaking cracks in concrete. Taking SAFT/TFM for comparison, crack depth and shape are better determined using the HSTFM-based analysis without the much larger numerical overhead necessary for methods like Reverse-Time Migration^51^ (RTM). The experimental database for HSTFM could simply be doubled by changing the instruments from half to full matrix capture. However, practicing both TFM and HSTFM techniques increases confidence in the detection and sizing of cracks.

Although the imaging results of both simulations and experiments appear consistent, there is still space to explore and optimize the performance of the HSTFM technique in critical conditions like branched cracks in confined regions. Owing to the limited resolution capabilities of the HSTFM technique, a single, optimum instrument position was used in this study to image the crack. The depth/amplitude plots presented are one of the basic analytical attempts to approximate crack sizes. The authors also view potentially open research areas in the directions of the complementing Half-Skip travel paths, factors affecting the resolution capabilities of half-skip imaging, and image-based sizing of cracks.

## Data Availability

A set of binary ultrasonic raw data sets of A1040 MIRA, taken on the concrete blocks, including the MATLAB script for their opening, are available from the corresponding author upon reasonable request.
